# Engineering Interfacial Integrity with Hydrolytic-Resistant, Self-Reinforcing Dentin Adhesive

**DOI:** 10.3390/ijms25137061

**Published:** 2024-06-27

**Authors:** Erhan Demirel, Burak Korkmaz, Youngwoo Chang, Anil Misra, Candan Tamerler, Paulette Spencer

**Affiliations:** 1 Institute for Bioengineering Research, University of Kansas, 1530 W. 15th Street, Lawrence, KS 66045-7608, USA; korkmazb@ku.edu (B.K.); ctamerler@ku.edu (C.T.); 2 Department of Chemistry, Faculty of Science and Letters, Istanbul Technical University, Maslak, Istanbul 34469, Turkey; 3 Department of Chemical and Petroleum Engineering, University of Kansas, 1530 W. 15th Street, Lawrence, KS 66045-7608, USA; ywchang54@gmail.com; 4 Department of Civil and Environmental Engineering, Florida International University, Miami, FL 33174-1630, USA; anmisra@fiu.edu; 5 Department of Mechanical Engineering, University of Kansas, 1530 W. 15th Street, Lawrence, KS 66045-7608, USA; 6 Bioengineering Program, University of Kansas, 1530 W. 15th Street, Lawrence, KS 66045-7608, USA

**Keywords:** silane monomer, sol-gel reaction, dynamic mechanical analysis, interface study, short-chain monomer, Raman spectroscopy, dental adhesive

## Abstract

The leading cause of composite restoration failure is secondary caries, and although caries is a multifactorial problem, weak, damage-prone adhesives play a pivotal role in the high susceptibility of composite restorations to secondary caries. Our group has developed synthetic resins that capitalize on free-radical polymerization and sol-gel reactions to provide dental adhesives with enhanced properties. The resins contain γ-methacryloxypropyltrimethoxysilane (MPS) as the Si-based compound. This study investigated the properties of methacrylate-based resins containing methacryloxymethyltrimethoxysilane (MMeS) as a short-chain alternative. The degree of conversion (DC), polymerization kinetics, water sorption, mechanical properties, and leachates of MMeS- and MPS-resins with 55 and 30 wt% BisGMA-crosslinker were determined. The formulations were used as model adhesives, and the adhesive/dentin (a/d) interfaces were analyzed using chemometrics-assisted micro-Raman spectroscopy. The properties of the 55 wt% formulations were comparable. In the 30 wt% BisGMA formulations, the MMeS-resin exhibited faster polymerization, lower DC, reduced leachates, and increased storage and loss moduli, glass transition (T_g_), crosslink density, and heterogeneity. The spectroscopic results indicated a comparable spatial distribution of resin, mineralized, and demineralized dentin across the a/d interfaces. The hydrolytically stable experimental short-chain-silane-monomer dental adhesive provides enhanced mechanical properties through autonomous strengthening and offers a promising strategy for the development of restorative dental materials with extended service life.

## 1. Introduction

The cycle of repeated composite restoration replacements is a pernicious problem. Each replacement risks pulpal injury, increased loss of tooth structure, increased tooth weakness, and eventually tooth loss [1]. The problem is pervasive; nearly 70% of all composite restorations are replacements for failed resin restorations [2]. The leading cause of composite restoration failure is secondary caries [3], and the composite/tooth margins of Class II and V restorations are particularly vulnerable to secondary caries [4]. Investigators report that 80–90% of secondary caries are located at the gingival margin of Class II and V composite restorations [5]. Patients at a high risk for caries are highly susceptible to composite restoration failure [3].

Composite materials lack the inherent capacity to seal gaps at the interface between the restorative material and the tooth structure. In general, this limitation is addressed using a low viscosity adhesive that bonds the viscous composite to the tooth structure. The clinical protocol for adhesive application begins with the removal of diseased tissue. Following the removal of the diseased tissue, the classical three-step etch-and-rinse (E&R) adhesive system involves simultaneous etching of enamel and dentin with phosphoric acid, followed by primer application and finally, adhesive [6]. In the E&R strategy, the monomer must infiltrate the wet, demineralized dentin to form a stable bond [7]. The classical three-step E&R adhesive system uses primers containing hydrophilic monomers and solvents to displace water from the demineralized dentin (which is primarily collagen) [8]. The primer is intended to prepare the collagen matrix for infiltration of the solvent-free, hydrophobic adhesive [8].

The adhesive is intended to infiltrate and provide micromechanical interlocking to the dentin collagen while also sealing the composite/tooth interface to provide a barrier to noxious agents that will undermine the composite restoration [6,9]. The mouth, however, is a hostile environment—diverse chemical, physical, and mechanical forces present in the mouth can work synergistically and simultaneously to degrade the adhesive [7,8,10,11,12]. One example is the hydrolytic degradation of adhesive polymers in the presence of water [7,12]. The hydrophilic monomers experience high water sorption that leads to water movement through the bonded interface—this activity ultimately leads to large water-filled channels and rapid degradation of the adhesive [7]. These events can have a cascading effect, e.g., water leads to plasticization and leaching of the solubilized resin which increases the surface area allowing more water and enzymes to penetrate, leading to hydrolytic degradation of the adhesive bond [7,12].

Adhesive degradation initiates a cascade of events that leads to voids and crevices at the composite/tooth interface. The crevices are colonized by cariogenic bacteria such as *S. mutans*. The bacteria and bacterial byproducts work cooperatively and synergistically to accelerate adhesive erosion, destroy tooth structure, and create wider and deeper gaps at the composite/tooth interface [13]. These activities ultimately lead to a downward spiral that culminates in composite restoration failure [9].

Investigators have explored various strategies to develop durable, damage-resistant dental adhesives. These strategies include eliminating [14] or shielding vulnerable ester bonds with bulky branched functional groups [15,16], replacing ester groups with ether-based vinyl functional groups [17], and adding magnesium-doped hydroxyapatite crystals to adhesives [18]. Other investigators have explored reinforcing the adhesive/dentin (a/d) bond and increasing its resistance to damage through plant-derived proanthocyanidins [19,20,21,22]. Matrix metalloproteinase (MMP) inhibitors have been included in dental adhesives to increase resistance to enzymatic degradation [23,24,25]. Other strategies include the development of multifunctional adhesives that incorporate magnetic filler particles doped with chlorhexidine and functionalized with quaternary ammonium silane [10]. The objective of this novel strategy is to provide adhesives that resist degradation, inhibit bacterial growth, and offer enhanced bond stability [10]. Other strategies are focused on novel chemistries that could improve the properties of repaired composite restorations, e.g., spherosilicate molecules with multiple methacrylate groups to serve as coupling agents between the inorganic filler particles and the resin matrix [26]. These multifaceted approaches have led to advances, but composite restoration failure continues to impose significant challenges on the oral health of individuals in the USA and around the globe [4].

The various strategies reflect the need to address the composite/tooth interface vulnerability through multi-pronged approaches. These approaches must consider both the adhesive infiltration required to protect dentin collagen and a durable adhesive that resists the degradative elements present in the mouth.

A strategy that our group has found particularly promising is alkoxysilane-containing adhesives that provide polymers with enhanced hydrolytic-resistance and improved mechanical properties through intrinsic network reinforcement under wet conditions [27,28,29,30,31,32,33]. As part of our efforts to refine and optimize these adhesives, we have explored the mechanical, chemical, and physical properties of alkoxysilane monomers in combination with various photoinitiators [27], buffering agents [28], tethered functionalized peptides (to inhibit bacteria and remineralize damaged dentin) [34], hydrophilic [31], and hydrophobic [32] formulations. Recognizing that any change in the chemistry could impact the mechanical and physical properties as well as adhesive infiltration of wet dentin collagen, the objectives of this study were to: (1) analyze the effect of the chain length between the silane and methacrylate functional groups on the mobility, crosslink density, and cohesive strength of the adhesive; and (2) analyze the alkoxysilane-containing adhesives at the interface with dentin. The hypotheses tested were that the chain length between the silane and methacrylate groups would have: (i) no effect on the thermal stability, hydrolytic-resistance, and mechanical strength of the bulk material at different crosslinker concentrations; and (ii) no effect on adhesive/dentin interfacial integrity.

## 2. Results

**Figure 1:**
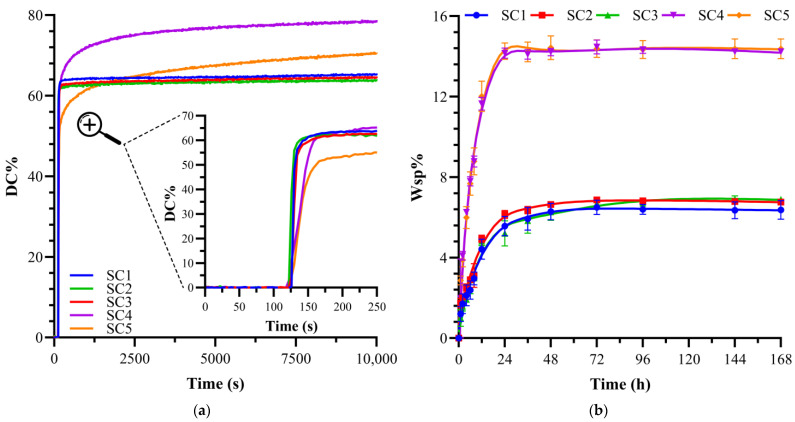
(**a**) Degree of conversion of SC formulations; (**b**) water sorption values of SC formulations.

Table 1 presents the degree of conversion (DC) and maximum polymerization rate (R_p_^max^/[M]) for resin formulations, i.e., SC1–SC5 (Figure 1a) and NE1 [32]. The compositions of the formulations SC1–SC5 and NE1 formulations are listed in Section 4.2. In brief, SC4 and SC5 are 15 wt% MPS and 15 wt% MMeS, respectively, in formulations with 30 wt% BisGMA-crosslinker. SC1–SC3 and NE1 are formulations with 55 wt% BisGMA-crosslinker. The DC of SC1, SC2, SC3 and NE1 are comparable at 65.4 ± 0.7, 63.9 ± 0.2, 64.5 ± 0.2, and 65.7 ± 0.8, respectively. Similarly, there was no statistically significant difference (*p* < 0.05) in the polymerization rates of SC1, SC2, SC3, and NE1 at 12.5 ± 1.6, 9.6 ± 2.3, 11.7 ± 1.3, and 11.1 ± 2.8, respectively. In comparison, the DC and polymerization rate of the formulations with reduced crosslinker concentrations, that is, 30 wt% BisGMA, were statistically significantly different. The DC of SC4 was significantly greater (*p* < 0.05) at 79.7 ± 0.2% compared to SC5 at 75.6 ± 0.2%. The polymerization rate (R_p_^max^/[M]) of SC4 is statistically significantly less (*p* < 0.05) at 2.06 ± 0.2 as compared to 2.78 ± 0.1 for SC5.

The solid copolymer resin discs experienced a gradual increase in water sorption, reaching a steady state between 36–48 h of storage at 37 °C (Figure 1b). There were no statistically significant differences in the water sorption for SC1, SC2, SC3, and NE1 at 6.37 ± 0.5, 6.76 ± 0.1, 6.90 ± 0.1, and 7.28 ± 0.5, respectively (Table 1). The formulations with reduced hydrophobic BisGMA crosslinker exhibited higher water sorption at 14.23 ± 0.1 and 14.38 ± 0.5 for SC4 and SC5, respectively. The higher water sorption reflected the relative increase in hydrophilicity as a result of the reduced BisGMA concentration.

**Figure 2:**
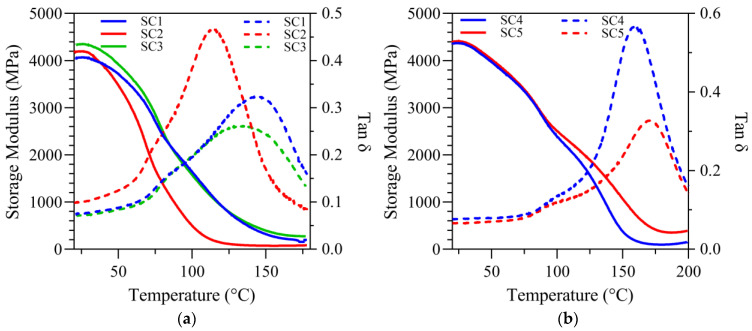
Under dry conditions: (**a**) tan δ vs. temperature curves and storage modulus vs. temperature curves for SC1, SC2, and SC3; (**b**) tan δ vs. temperature curves and storage modulus vs. temperature curves for SC4 and SC5.

**Figure 3:**
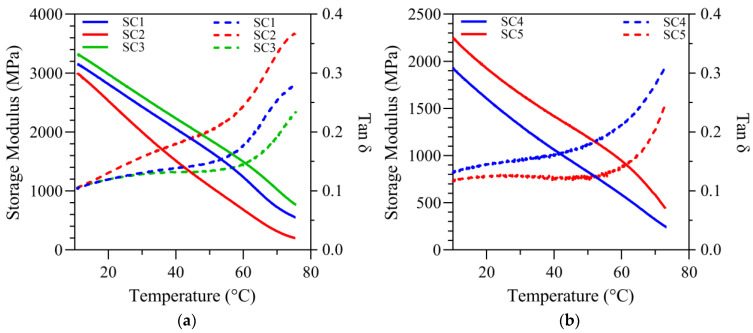
In submerged conditions (**a**) tan δ vs. temperature curves and storage modulus vs. temperature curves for SC1, SC2 and SC3; (**b**) tan δ vs. temperature curves and storage modulus vs. temperature curves for SC4 and SC5.

The dynamic mechanical properties of the resin formulations under both dry and wet conditions were evaluated rigorously and the results are presented in Figure 2 and Figure 3 and summarized in Table 2, Table 3 and Table 4. The tan δ versus temperature plots, highlighted in Figure 2 and Figure 3 (right *y*-axis), illustrate the glass transition temperatures (T_g_) of the formulations. Under dry conditions, the results demonstrated a clear difference between formulations with different crosslinker concentrations. Specifically, the T_g_ of the SC5 formulation (30 wt% BisGMA) was significantly higher (*p* < 0.05) at 170.3 ± 0.9 °C than the T_g_ (°C) values of SC1, SC2, SC3, and NE1 at 144.2 ± 5.9, 114.1 ± 2.5, 135.2 ± 13.6, and 159.8 ± 4.6, respectively. The T_g_ of SC5 was significantly higher (*p* < 0.05) than SC4 (159.6 ± 0.8 °C). The differences in T_g_ reflect the restricted mobility of the copolymer in the SC5 formulation (Figure 2b).

The mechanical property investigations conducted under dry conditions for SC1, SC2, and SC3 covered the temperature range of 20–180 °C. This range was extended to 220 °C for SC4 and SC5, reflecting their adaptation to elevated T_g_ values under dry conditions. Owing to the operational temperature limit imposed by the three-point bending submersion clamp, the mechanical property testing under wet conditions was constrained to a maximum of 70 °C.

At a uniform temperature of 37 °C under dry conditions, the storage moduli (GPa) for SC1 through SC5 formulations were comparable at 3.96 ± 0.28, 3.97 ± 0.10, 4.24 ± 0.35, 4.21 ± 0.24, and 4.40 ± 0.18, respectively. In comparison, at 70 °C, the storage modulus values (GPa) showed notable differences among some formulations (3.00 ± 0.20, 2.09 ± 0.16, 3.15 ± 0.28, 3.47 ± 0.19, and 3.62 ± 0.15 for SC1, SC2, SC3, SC4, and SC5, respectively), with significant differences recorded between pairs SC1–SC2, SC1–SC5, SC2–SC3, SC2–SC4, SC2–SC5, and SC3–SC5 (*p* < 0.05). The other combinations showed no significant differences.

There were pronounced differences between the formulations at temperatures beyond the threshold of 175 °C within the rubbery modulus plateau. Noteworthy is the absence of a significant difference between SC2 and SC4 at this elevated temperature range (>175 °C), where the values are 0.08 ± 0.00 GPa and 0.10 ± 0.01 GPa, respectively. In comparison, the rubbery moduli (GPa) of SC1, SC3, and SC5 (0.20 ± 0.02, 0.27 ± 0.06, and 0.36 ± 0.02, respectively) are statistically significantly greater (*p* < 0.05) than those of SC2 and SC4 (Table 2).

Under wet conditions, a similar trend of decreasing storage modulus values from 37 to 70 °C was recorded for all formulations, mirroring the pattern observed under dry conditions. The storage modulus values (GPa) at 37 °C under wet conditions are 2.14 ± 0.20, 1.95 ± 0.28, 2.34 ± 0.12, 1.14 ± 0.08, and 1.49 ± 0.05 for SC1, SC2, SC3, SC4, and SC5, respectively. In comparison, at 70 °C the storage modulus values (GPa) are 0.69 ± 0.12, 0.46 ± 0.16, 1.02 ± 0.13, 0.32 ± 0.03, and 0.57 ± 0.02 for SC1, SC2, SC3, SC4, and SC5, respectively. The results are presented in Table 3.

Table 4 presents the calculated crosslink density (ζ) values (Pa^−1^K) [35,36] and the full width at half maximum (FWHM) for SC1–SC5. A markedly lower ζ value for SC5 (0.52 ± 0.0 × 10^−6^) in comparison to SC1, SC2, SC3, and SC4 (2.32 ± 0.2 × 10^−6^, 5.61 ± 0.3 × 10^−6^, 1.69 ± 0.3 × 10^−6^, and 1.97 ± 0.1 × 10^−6^, respectively) reflected higher crosslink density in SC5, despite a reduced ratio of crosslinker. The FWHM value of SC5 (66.06 ± 2.1) was significantly greater than SC4 (48.62 ± 1.1), indicating a more heterogeneous polymer network even at equivalent crosslinker concentrations. The FWHM values for SC3 and SC1 are 96.23 ± 5.8 and 86.72 ± 5.2, respectively, indicating an increase in polymer network heterogeneity. The FWHM of SC3 (96.23 ± 5.8) was statistically significantly (*p* < 0.05) greater than SC1 (86.72 ± 5.2) and SC2 (64.51 ± 2.8).

To enhance the sol-gel reaction, the disc samples were incubated in ultrapure water for seven days. 

Table 5 presents the average amounts of HEMA leached in the prewash solutions. Specifically, the amounts of HEMA leached (μg/mL) in the prewash were 20.66 ± 1.74, 40.62 ± 0.02, and 27.16 ± 0.84 for SC3, SC4, and SC5, respectively. The percentages (wt%) of leached HEMA based on the total HEMA in the corresponding formulation were 0.30 ± 0.02, 0.31 ± 0.00, and 0.20 ± 0.01 for SC3, SC4, and SC5, respectively. The relatively low levels of HEMA leachate in the prewash solutions suggest limited unreacted monomers in the dark-cured and water-incubated samples.

**Figure 4:**
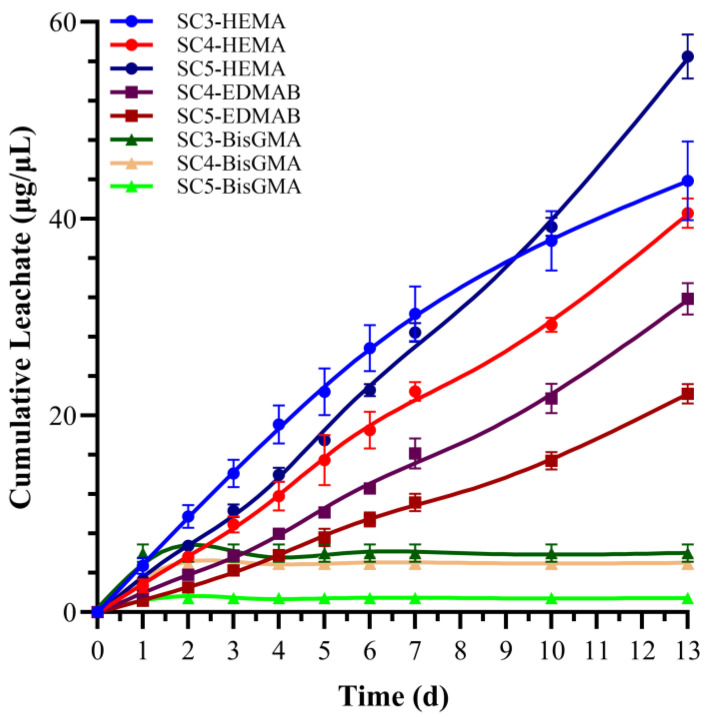
Average cumulative leachate concentrations of components from SC3, SC4, and SC5.

Figure 4 shows the average cumulative concentrations of leachates from the samples stored in ethanol at room temperature (23 ± 2 °C). The ethanol solutions were analyzed for the following leachates: MMeS (SC3 and SC5), MPS (SC4), HEMA, EDMAB, and BisGMA (Table 6). MMeS and MPS leachates were not detected in the ethanol solutions. The cumulative leachate values, determined through chromatographic peak intensity comparisons with the standard solution calibration curves, revealed that the leachates of HEMA, EDMAB, and BisGMA from SC5 were significantly different (*p* < 0.05). Specifically, the average cumulative concentrations (μg/mL) of EDMAB leached from SC4 and SC5 were 31.86 ± 1.6 and 22.22 ± 1.0, respectively. The weight percentages (wt%) based on the total EDMAB in the corresponding formulation are 25.49 ± 1.3 and 17.78 ± 0.8 for SC4 and SC5, respectively. EDMAB leached from SC5 was statistically significantly less (*p* < 0.05) than EDMAB leached from SC4 (Table 7). HEMA leachate levels (μg/mL) were 43.87 ± 4.0, 40.57 ± 1.5, and 56.49 ± 2.24 for SC3, SC4, and SC5, respectively. The weight percentages (wt%) of leached HEMA, based on the total HEMA in the corresponding formulation, are 0.63 ± 0.1, 0.31 ± 0.0, and 0.43 ± 0.0 for SC3, SC4, and SC5, respectively (Table 7). BisGMA leachate levels were significantly lower than those of HEMA. BisGMA leachate levels (μg/mL) were 6.00 ± 0.9, 5.01 ± 0.6, and 1.44 ± 0.2 for SC3, SC4, and SC5, respectively (Table 6). Weight percentages (wt%) of leached BisGMA were 0.04, 0.07, and 0.02 for SC3, SC4 and SC5, respectively (Table 7). The kinetic degradation behavior of the ethanol-stored samples from days 0 to 13 is presented in Figure 4. A sharp decline in the quantity of leached species was observed following the initial week of ethanol aging, and the rate of leachate accumulation decreased after the tenth day.

**Figure 5:**
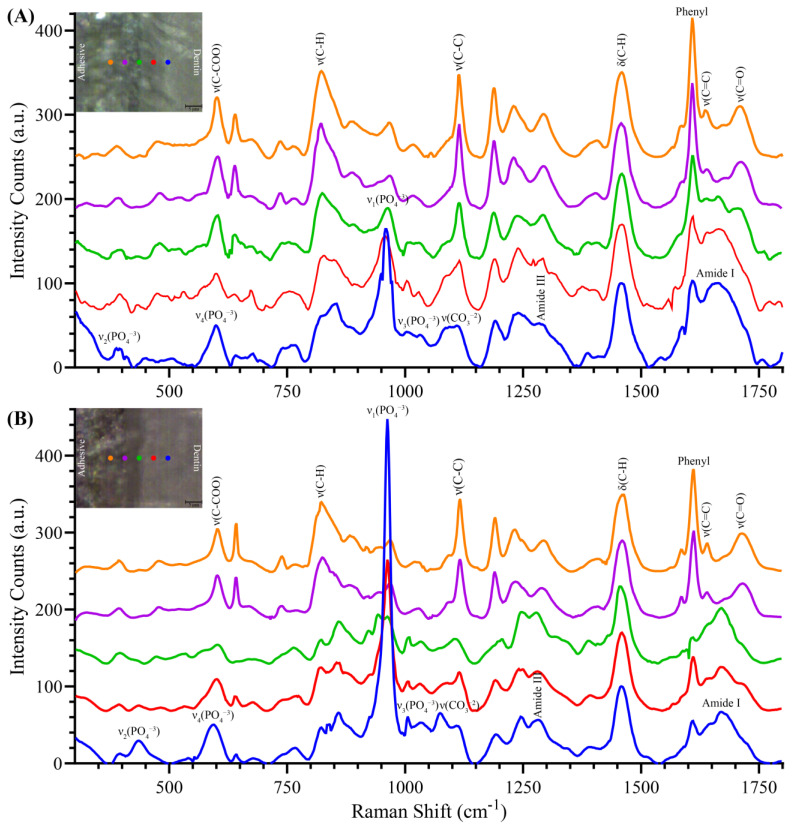
Spectra overlay for (**A**) SC4–NE1 and (**B**) SC5–SC3 depicting Raman spectra at various depths starting from adhesive section (orange line), adhesive-dentin interface (green line) and dentin section (blue line).

Two-dimensional XY Raman imaging was performed from the scan areas of the a/d interface specimens shown in the light micrographs (Figure 5A,B). Spectra were acquired at points across adhesive, demineralized dentin (collagen), and mineralized dentin. Spectral features associated with the adhesive appeared at 601 cm^−1^ (C–COO), 822 cm^−1^ (ν CH_2_), 1112 cm^−1^ (ν C–C), 1463 cm^−1^ (CH def), 1610 cm^−1^ (phenyl), 1638 cm^−1^ (ν C=C), and 1710 cm^−1^ (ν C=O). The spectral features assigned to dentin collagen were 1670 cm^−1^ (C=O, amide I), 1460 cm^−1^ (CH_2_ wagging), and doublet bands from 1250 to 1277 cm^−1^ (C–N stretching and N–H bending, amide III). The Raman spectral features associated with dentin mineral were 435 cm^−1^ (ν_2_ PO_4_^−3^), 593 cm^−1^ (ν_4_ PO_4_^−3^), 960 cm^−1^ (ν_1_ PO_4_^−3^), 1037 cm^−1^ (ν_3_ PO_4_^−3^), and 1074 cm^−1^ (CO_3_^−2^).

**Figure 6:**
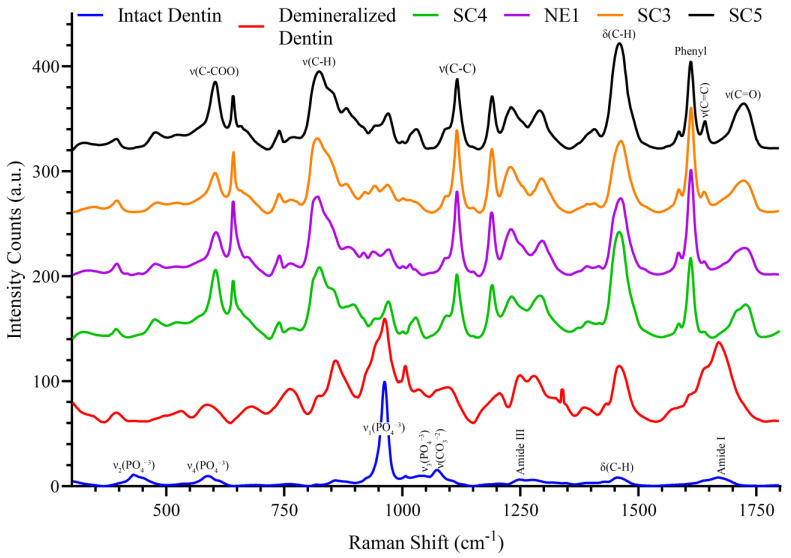
Reference Raman spectra of formulations SC5, SC3, NE1, SC4, partially demineralized, and intact dentin (top to bottom).

The reference spectra of the formulations that were part of the two-phase adhesive systems are shown in Figure 6. SC4 was applied to the etched dentin surface, followed by treatment with NE1, and the adhesive was photopolymerized, as described in Section 4.8. SC5 was applied to the etched dentin, followed by treatment with SC3, and the adhesive was photopolymerized, as described in Section 4.8. For ease of comparison, the reference spectra of the partially demineralized and mineralized dentin are presented in Figure 6.

**Figure 7:**
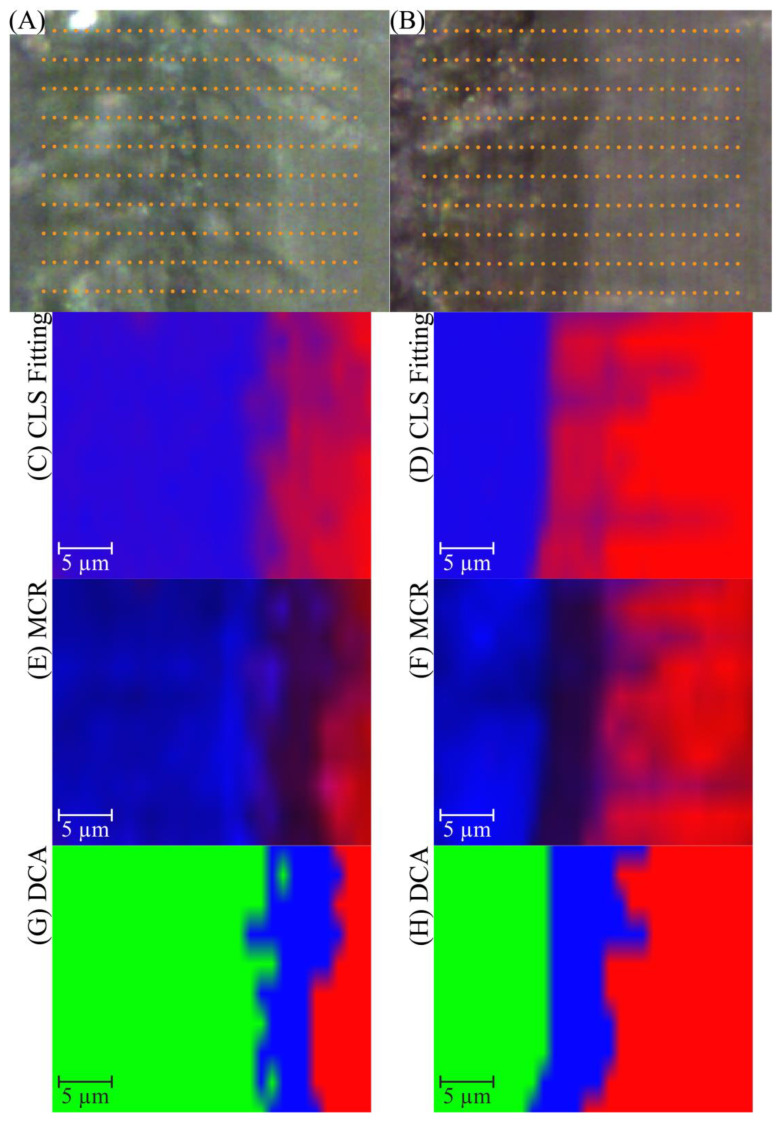
Raman microscopy images and multivariate analysis results for adhesive/dentin (a/d) interface specimens treated with different adhesive formulations. (**A**,**B**) Optical microscopy images, (**C**,**D**) CLS fitting maps, (**E**,**F**) MCR analysis maps, and (**G**,**H**) DCA maps for the SC4–NE1 and SC5–SC3 samples, respectively.

The a/d interface specimens were analyzed using micro-Raman spectroscopy to determine the spatial distribution of adhesive, collagen (demineralized dentin), and mineral components. The spectral data were processed using the multivariate analysis (MVA) module in LabSpec 6, employing classical least squares (CLS) fitting, multivariate curve resolution (MCR), and divisive clustering analysis (DCA) (Figure 7).

Confocal microscopy images of the a/d interface specimens treated with different adhesive formulations are shown in Figure 7A,B. Image A is associated with the SC4–NE1 sample, whereas image B is associated with the SC5–SC3 sample. Both images display intricate morphological features at the a/d interface, showing distinct patterns that highlight structural differences in the samples. The orange dots superimposed on the optical images served as a spatial mapping grid for subsequent Raman spectroscopy data acquisition.

The CLS fitting maps for SC4–NE1 and SC5–SC3 are illustrated in Figure 7C,D, respectively. These maps depict the chemically distinct components of the Raman spectral data, represented by different colors, following the decomposition achieved by the CLS approach. Reference spectra for both dentin and adhesive were used to model the acquired data. The maps provide a visual representation of the spatial distribution and relative abundance of adhesive components within dentin. The SC4–NE1 system demonstrated a distinct distribution pattern compared to the SC5–SC3 system, suggesting differences in adhesive infiltration and interaction with the dentin substrate. In the SC4–NEI system, the prominent differentiation between regions is indicated by blue and red colors, which represent the distribution of the adhesive and dentin components, respectively. In contrast, the SC5–SC3 sample (Figure 7D) showed a more homogeneous distribution, with the CLS mapping revealing a clear demarcation between the two distinct regions across the a/d interface.

MCR (Figure 7E,F) was performed to decompose the spectral data into pure component spectra, facilitating a detailed understanding of the distribution of individual chemical species within the Raman maps and a more nuanced understanding of the chemical composition at the a/d interface. The MCR method improves the resolution of chemical species distributions by resolving overlapping spectral features. For SC4–NE1 (Figure 7E), the spatial map exhibited regions of mixed chemical compositions, indicating areas where the interdiffusion of adhesive and dentin occurred. Conversely, the SC5–SC3 sample (Figure 7F) displayed distinct regions with reduced intermixing, suggesting enhanced phase separation between the components. The depth of the adhesive infiltration was measured from the generated maps. Based on these measurements, the depth was 4 ± 0.5 µm for SC4–NE1 and 5 ± 0.4 µm for SC5–SC3 suggesting increased adhesive infiltration with the SC5–SC3 system.

Divisive clustering analysis (DCA) (Figure 7G,H) was employed to classify and group the acquired spectra into distinct clusters. The iterative process divides the spectra into three components, resulting in a class membership for each spectrum. The average spectra for each class were calculated, and these results were used to determine the component distribution within the Raman images. The DCA maps showed a clear segmentation of the adhesive, collagen, and mineral components of dentin, providing a comprehensive overview of the spatial arrangement of these materials at the interface. These clusters indicated a heterogeneous distribution of chemical components at the interface with the SC4–NE1 sample. In contrast, the DCA map for the SC5–SC3 sample (Figure 7H) revealed a more coherent pattern with larger contiguous regions, suggesting a more uniform interaction between the adhesive and dentin components. A similar measurement, with the MCR analysis, was conducted to obtain a better understanding of the depth of adhesive infiltration with SC4–NE1 and SC5–SC3, which was found to be 4 ± 1 and 6 ± 1 µm, respectively.

## 3. Discussion

### 3.1. Polymerization Behavior: Role of Crosslinker Concentration

Polymerization behavior (degree of conversion (DC) and maximum polymerization rate) plays an important role in determining the quality of the a/d interfacial bond. The conversion of the C=C bond is rarely complete in the multifunctional methacrylate monomers. The lack of complete conversion was due to the limited mobility of the propagating free radical species in the crosslinked network. The results of our earlier studies on alkoxysilane-containing adhesives indicated that (1) with the three-component photoinitiator system, the amine radicals initiate the polymerization of methacrylate and are oxidized by iodonium to form H^+^ protons that can catalyze the hydrolysis of the methoxysilyl moieties; (2) the limited mobility of the polymer side chains in the highly crosslinked methacrylate matrix retarded the condensation of silanol groups; and (3) the hydrolysis-condensation rate was relatively low compared to free-radical polymerization [27].

The current investigation explored the impact of crosslinker concentration on organosilanes with different chain lengths between silane and methacrylate functional groups. The FTIR results indicated that the difference in the organosilanes (MPS and MMeS) had a negligible effect on DC and maximum polymerization rate in formulations with 55 wt% BisGMA crosslinker. Early copolymer network stiffening and reduced mobility of free radicals in the polymethacrylate-based network structure, led to a lower DC and faster polymerization rate with the 55 wt% BisGMA formulations. In comparison, the FTIR results for the 30 wt% BisGMA formulations indicated an increased degree of conversion, decreased polymerization rate, and statistically significant differences between the organosilanes. These results can be attributed to several factors, including: (1) the reduced crosslinked methacrylate matrix led to enhanced mobility of the side chains and propagation of free radical species; (2) the reduced crosslinker concentration inhibited early copolymer network stiffening, which could interfere with free-radical polymerization and sol-gel reactions; and (3) the reduced concentration of the hydrophobic BisGMA crosslinker facilitated the delivery of water to the organosilanes, which led to more effective sol-gel reactions.

### 3.2. Water Sorption

The water sorption results indicated a rapid increase in sorption behavior during the initial 24 h of storage at 37 °C. Water sorption plateaued at 36–48 h. The results indicated comparable water sorption with both organosilanes (MPS and MMeS) in formulations containing 55 and 30 wt% BisGMA. However, the 30 wt% BisGMA formulations had a twofold increase in retentive capacity for water compared to the 55 wt% BisGMA formulations. The higher water sorption was attributed to a relative increase in hydrophilicity in the 30 wt% BisGMA formulations.

### 3.3. Dynamic Mechanical Analysis

DMA tests were carried out using both standard and submersion three-point bending methods. The mechanical properties of the formulations tested under dry conditions are presented in Figure 2 and summarized in Table 2. The storage moduli are comparable at a constant temperature of 37 °C under dry conditions. In comparison, at 70 °C under dry conditions, notable differences were observed among some of the formulations. These differences may be related, in part, to DC. Investigators have correlated DC with mechanical properties [15,37,38]. The DC of the 55 wt% BisGMA formulation containing two silane monomers (SC2, MPS, and MMeS at 10 and 5 wt%) was lower than that of the other formulations, which could contribute to the lower storage moduli at 70 °C under dry conditions.

Young et al. reported increased heterogeneity as the concentration of the crosslinking agent increased within a copolymer system [37]. With the exception of SC2, the full width half maximum values indicated increased heterogeneity with 55 wt% BisGMA compared to 30 wt% formulations.

Usually, reduced molecular mobility is inferred from the increased glass transition temperature. The glass transition temperature of the SC5 formulation containing 30 wt% BisGMA and 15 wt% short-chain silane monomer (MMeS) was significantly higher than that of the 55 wt% BisGMA formulations. The glass transition temperature depends on several factors, including the DC, monomer viscosity, and functionality. As shown in Figure 1, the DC of the 30 wt% BisGMA formulation was greater than that of the 55 wt% formulation. The fast polymerization rate of 55 wt% BisGMA leads to early copolymer network stiffening, which interferes with free radical polymerization and sol-gel reactions.

The comparison of the 30 wt% BisGMA formulations revealed a significantly higher glass transition temperature with SC5 (15 wt% short-chain monomer, MMeS) than with SC4 (15 wt% MPS). The differences in T_g_ values reflect the restricted mobility of the copolymer in the SC5 formulation. The results suggest that differences in the chain length between the silane and methacrylate functional groups can significantly affect the mobility.

The rubbery modulus values of SC5 (30 wt% BisGMA and 15 wt% MMeS) were significantly higher than those of SC4 (30 wt% BisGMA and 15 wt% MPS) (Table 2). These differences suggest additional crosslinks in SC5 formulations. The relative increase in the crosslink density of SC5 suggests that the chain length between the silane and methacrylate functional groups leads to additional covalent bond formation because of the heterocondensation reaction of the HEMA/BisGMA hydroxyl groups and silanols.

Specimens for submersion testing were kept in water until they reached a constant mass. The water saturated specimens were tested using a water submersion clamp over a temperature range of 10–70 °C at a heating rate of 1.5 °C/min and a frequency of 1 Hz. We postulated that using this approach, the results were more representative of the mechanical responses in wet and oral environments.

The storage moduli of the formulations measured by the water submersion method were significantly lower than the values measured under dry conditions. The storage modulus values under wet conditions showed a decreasing trend with increasing temperature. The difference in the storage moduli under wet and dry conditions was attributed to the plasticizing effect of water. Similar differences were noted in our previous studies [27,31,32].

The storage modulus values of the 55 wt% BisGMA formulations were comparable under wet conditions. Under wet conditions, the storage moduli of the 30 wt% BisGMA were lower than those of the 55 wt% formulations at both 37 °C and 70 °C. The difference in the storage moduli under wet conditions is attributed to the increased hydrophilicity of the 30 wt% formulations. The increased hydrophilicity led to increased water sorption with the concomitant plasticization of the polymer network. Although not statistically significant, the storage moduli of SC4 were lower than those of SC5 at both 37 and 70 °C. This difference was attributed to the additional crosslinks in SC5 formulations.

Interestingly, storage modulus values, under both dry and wet conditions, of the 15 wt% MMeS (SC3) are greater than the 15 wt% MPS (NE1) in the 55 wt% BisGMA formulations. This difference is particularly apparent at 70 °C under wet conditions. At this temperature, and under wet conditions, the storage modulus values of the formulations containing the short-chain monomer (MMeS) were more than three times greater than those of the MPS-containing formulations. This difference is attributed to the additional crosslinks in SC3 compared to NE1.

### 3.4. HPLC Analysis

Ethanol was used as the solvent to enhance the solubility of the hydrophobic components and accelerate their diffusion from the polymers. The mechanical properties of SC1 and SC2 were inferior to those of the other formulations; therefore, SC1 and SC2 were not included in the leachate study. The results indicated that over 25% of the co-initiator EDMAB was leached from SC4. Based on our previous studies with MPS-containing formulations, this relatively high concentration of leachate was due to EDMAB molecules trapped in the crosslinked network [27]. The significantly lower concentration of EDMAB (>17%) leached from SC5 could be attributed to its higher crosslinking density. Although the results were not significantly different, the weight percentage of HEMA leached from SC3 (55 wt% BisGMA and 15 wt% MMeS) was greater than that leached from SC4 and SC5. These differences are related to the lower DC of SC3 compared to SC4 and SC5. The weight percentage of the leached BisGMA was significantly lower than that of HEMA. The weight percentage of BisGMA leached from SC4 was more than threefold greater than that of SC5. This difference was related to the increased crosslinking density of SC5. The two-fold greater weight percentage of BisGMA leached from SC3 compared to SC5 is related to the lower DC and crosslink density of SC3.

### 3.5. Chemometrics-Assisted Raman Analysis of the Adhesive/Dentin (a/d) Interface

As noted in the text associated with Figure 6, the formulations were used as a part of a two-phase adhesive system. Formulations with 30 wt% BisGMA (SC4 and SC5) were applied to the etched dentin surface. Formulations with 55 wt% BisGMA were applied to the treated surfaces, that is, etched dentin–SC4–NE1 and etched dentin–SC5–SC3, and photopolymerized. The three-step bonding procedure used in the a/d investigation is considered the best approach for determining the performance of individual components [8]. Furthermore, the classical three-step E&R adhesive system, which involves conditioning dentin and enamel with phosphoric acid (conditioner) followed by primer application and finally, adhesive, is the gold standard for dental adhesion [6]. Recent reports indicate that hydrophobic-rich coatings produce a two-fold increase in the endurance limit of the interfacial resin–dentin bond [12] and E&R adhesives provide higher micro-tensile bond strength than self-etch adhesive systems [39,40].

The specimens were analyzed using a combined Raman spectroscopy and chemometrics approach [41]. The approach correlates physical properties to analytical data by extracting important details that are often hidden in spectra acquired from complex, heterogeneous samples [42,43,44,45,46,47,48,49] such as the a/d interface.

In this study, the CLS, MCR, and DCA techniques provided robust analysis of the a/d interface. The results from the cluster analyses illustrated the complex nature of the a/d interface and highlighted the distinct effects of different adhesive formulations on the distribution and interaction of various components. CLS fitting enabled precise quantification of adhesive and dentin components, whereas MCR allowed for the resolution of pure spectra from mixed data [48], facilitating the identification of relative regions. DCA offers a clear classification of the spectral data, enhancing the understanding of the spatial distribution of adhesive, collagen, and mineral components. These multivariate methods have demonstrated their efficacy in analyzing complex datasets, providing detailed insights into the molecular interactions and distribution patterns of the components that constitute the heterogeneous a/d interface.

The observed differences between the SC4–NE1 and SC5–SC3 systems highlight the impact of adhesive formulation on dentin infiltration and interfacial integrity. The SC4–NE1 formulation displayed a greater level of intermixing but with a shallower depth of infiltration compared to the SC5–SC3 sample. Conversely, the SC5–SC3 sample presented more defined compositional boundaries, suggesting a distinct separation of phases, but with deeper adhesive infiltration.

## 4. Materials and Methods

### 4.1. Materials

The following monomers: 2-hydroxyethyl methacrylate (HEMA), bisphenol A glycerolate di-methacrylate (BisGMA), and γ-methacryloxypropyl trimethoxy silane (MPS) were purchased from Sigma-Aldrich (St. Louis, MO, USA) and used as received without further purification. The photoinitiators: diphenyliodonium hexafluorophosphate (DPIHP), camphorquinone (CQ), ethyl-4-(dimethylamino) benzoate (EDMAB) were also purchased from Sigma-Aldrich (St. Louis, MO, USA). The short-chain monomer, (trimethoxysilyl)methyl methacrylate (MMeS) was acquired from Ambeed Chemicals (Arlington Hts, IL, USA) and used as received without further purification. All other chemicals were of reagent grade and used as received without further purification.

**Figure 8:**
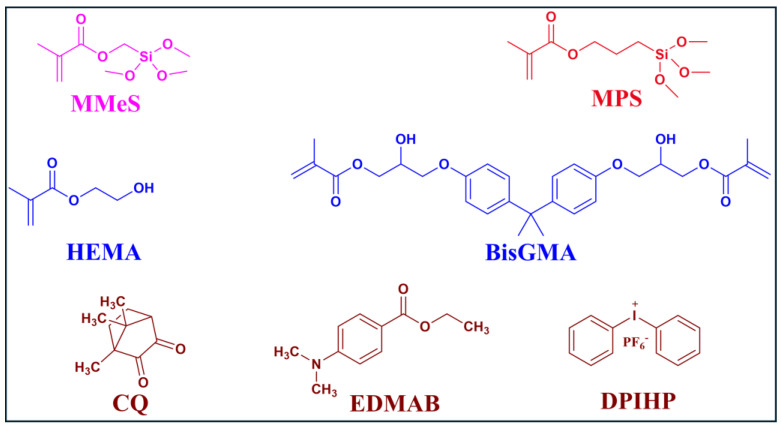
Structures of the monomers and photoinitiators in the formulations.

The chemical structures of the monomers and photoinitiators are shown in Figure 8.

### 4.2. Preparation of Adhesive Formulations

A three-component photoinitiator system (CQ-EDMAB-DPHIP (0.5/0.5/1 wt/wt/wt)) was used for each resin formulation [32]. All the mixtures were prepared under amber light in brown glass vials [27]. This procedure was necessary to avoid premature polymerization. For each formulation, HEMA and the organosilanes MMeS (for SC1, SC2, SC3, and SC5) and MPS (for NE1 [32], SC1, SC2, and SC4) were added to amber vials, photoinitiators were added, and the solutions were mixed thoroughly to obtain homogeneous mixtures. BisGMA was added to the mixture and the formulations were stirred and shaken for 24 h at room temperature (23 ± 2 °C).

HEMA/BisGMA/MMeS (28/55/15, SC3) and HEMA/BisGMA/MMeS (53/30/15, SC5) were used as the boundary conditions for the experimental formulations. HEMA/BisGMA/MPS/MMeS (28/55/5/10, SC1) and HEMA/BisGMA/MPS/MMeS (28/55/10/5, SC2) formulations were used to study the effects of the two silane monomers. HEMA/BisGMA/MPS (28/55/15, NE1 [32]) and HEMA/BisGMA/MPS (53/30/15, SC4) formulations were used as control formulations.

The formulations used are listed in Table 8. For ease of comparison, the compositions and properties of NE1 [32] are included in the relevant tables.

### 4.3. Real-Time Double Bond Conversion and Maximum Polymerization Rate

Fourier transform infrared spectroscopy (FTIR) was used to determine the degree of conversion (DC) of monomer to polymer and the polymerization rate [32,36]. Utilizing a Frontier FTIR Spectrometer (Perkin-Elmer, Shelton, CT, USA) with a spectral resolution of 4 cm^−1^ across a wavenumber range of 650–4000 cm^−1^, the photopolymerization process was recorded in situ in real time. This instrument is equipped with Spectrum TimeBase v3.0 software (Perkin-Elmer, Shelton, CT, USA), facilitating continuous spectral acquisition, thus enabling the quantification of DC over time. The transformation of the methacrylic C=C double bonds was monitored by analyzing the ratio of the absorbance at 1637 cm^−1^ (methacrylic bond) to that at 1714 cm^−1^ (indicative of carbonyl groups). DC was computed using the formula DC = (1 − R_p_/R_R_) × 100, where R_p_ represents the post-polymerization band ratio and R_R_ denotes the pre-polymerization band ratio [32]. The average of the final 30 DC measurements, which stabilized at a plateau, was recorded as DC.

For the experimental preparation, 5–10 μL of the adhesive mixture was applied to the crystal of an attenuated total reflectance (ATR) accessory (Universal ATR Sampling Accessory, Perkin-Elmer, Shelton, CT, USA). To mitigate the interference from atmospheric oxygen and moisture, the adhesive was shielded with a Mylar film. Following an initial analysis period of 120 s, the sample was irradiated with a commercial visible light curing unit (Spectrum 800, Dentsply, Charlotte, NC, USA) at a peak wavelength of 488 nm [50] and an intensity of 550 mW/cm^2^ for 40 s, during which the infrared spectra were collected continuously for approximately three hours. Each adhesive formula underwent two to three separate measurements.

The polymerization kinetics were determined by calculating the maximum rate of conversion (R_p_^max^) derived from the first derivative of DC over time. The values of DC and R_p_^max^ are presented in Table 1 [36].

### 4.4. Water Sorption

Round disc samples (1.2 mm × 4 mm diameter) were prepared for leachate and water sorption studies. Homogeneous mixtures of adhesives were added to cylindrical 1 mL syringes (BD, Becton, Dickinson and Company, Franklin Lakes, NJ, USA). Considerable effort was required to avoid introducing air bubbles when filling syringes. The filled and sealed syringes were placed in an LED light-curing system with the following characteristics: wavelength 400–500 nm, 100 mW/cm^2^ irradiance (LED cure-dome, [51,52], Prototech, Portland, OR, USA) to undergo visible light photopolymerization for 40 s. Following light polymerization, the syringes were stored in the dark for a minimum of 48 h. After light polymerization and dark curing, disc samples were prepared by sectioning the syringes to the intended thickness using a Isomet 1000 Precision Saw (Buehler, Lake Bluff, IL, USA) equipped with a water-cooled diamond disk. The resulting disc samples were prewashed by submerging in 1 mL of water for seven days at 37 °C. The water from the prewash was collected, and the species that leached in the prewash were analyzed using high-performance liquid chromatography (HPLC) (Described Section 4.6). Following the prewash, the samples were thoroughly dried under vacuum until a constant mass, m1, was achieved. After the mass plateaued, five-disc samples for each formulation were submerged in 2 mL of ultrapure water and weighed at the following time intervals: 0, 1, 2, 4, 6, 8, 12, 24, 36, 48, 72, 96, and 120 h, or until they reached a constant mass, m_2_. The water sorption was calculated using the following equation:

(1) $$W_{sp}\%=\frac{m_{2}-m_{1}}{m_{1}}\times 100$$

### 4.5. Dynamic Mechanical Analysis (DMA)

The viscoelastic behavior of the five resin formulations was evaluated using a dynamic mechanical analyzer (DMA Q800, TA Instruments, New Castle, DE, USA), which features a liquid nitrogen-based cooling system for precise temperature control. Vacuum-dried rectangular beam specimens were analyzed using a standard three-point bending clamp, whereas specimens conditioned under water immersion were assessed using a three-point bending submersion clamp. To prepare the rectangular beam specimens, 30–40 μL of the resin formulation was injected into borosilicate glass tubes (Vitrocom Technical Glass, 8100 Square VitroTubes™, Mountain Lakes, NJ, USA) at room temperature, followed by photopolymerization in an LED light-curing system (LED cure-dome, 100 mW/cm^2^ irradiance, Prototech, Portland, OR, USA) for 40 s. The samples were post-cured in the dark for one hour. Following post-curing, the samples were demolded and subjected to hydrolytic conditioning in water at 37 °C for seven days, followed by 48–72 h of aging at the same temperature to promote condensation reactions [36]. For the experimental setup, a total of ten beam specimens per formulation were randomly divided into two groups for comparative analysis under dry and wet conditions, respectively.

Beam specimens designated for drying were exposed to vacuum at 37 °C until a constant mass was achieved. This process requires a minimum of 96 h. Dynamic mechanical analysis was conducted over a temperature range of 20–180 °C for formulations SC1, SC2, and SC3 at a heating rate of 3 °C/min and a frequency setting of 1 Hz. The temperature range was extended to 220 °C for both SC4 and SC5. Specimens prepared for wet condition testing were saturated in ultrapure water at 37 °C until equilibrium mass was reached, followed by analysis over a temperature spectrum of 10–75 °C, with a heating rate adjusted to 1.5 °C/min and a frequency of 1 Hz. A uniform support span of 10 mm was used for all the tests [33].

### 4.6. Leachable Study: Prewash and Prewashed Samples Aged in Ethanol

Disk samples (n = 3) of SC3, SC4, and SC5 were prepared following the protocol outlined in Section 4.4 (Water Sorption). SC1 and SC2 were not analyzed in the leachate study because the mechanical properties of these formulations were inferior to those of SC3, SC4, and SC5. The disc samples were prewashed in water for seven days at 37 °C. The water from the prewash was collected, and the species that leached during the prewash were analyzed using HPLC, as described below.

Once a constant mass was attained by the dried disc samples, they were immersed in 1 mL of HPLC-grade ethanol (HPLC-Grade). The ethanol solutions were periodically sampled at 24 h intervals during the initial week and subsequently at 72 h intervals beyond the seventh day, with each sampling followed by replenishment with fresh ethanol. The eluted substances in the ethanol were analyzed quantitatively by HPLC using a LC-2010C HT system (Shimadzu, Lenexa, KS, USA) complemented by EZstart software version 7.4 SP2, and a 250 × 4.6 mm column filled with 5 μm C-18 silica (Luna, Phenomenex Inc., Torrance, CA, USA). HPLC analysis was performed using a mobile phase of acetonitrile and water containing 0.1% trifluoroacetic acid (TFA), employing gradient elution from 15/85 (*v*/*v*) to 100/0 (*v*/*v*) over 56 min. Operational parameters were set to a flow rate of 1 mL/min, UV detection at 208 nm, an injection volume of 20 μL, and a column temperature of 40 °C. Calibration of the column was executed with standard solutions of BisGMA, HEMA, MPS, MMeS, and EDMAB, generating calibration curves for BisGMA (2.5–250 μg/mL, R^2^ = 0.9995), HEMA (2.5–250 μg/mL, R^2^ = 0.9998), MPS (2.5–125 μg/mL, R^2^ = 0.9997), MMeS (2.5–250 μg/mL, R^2^ = 0.9996) and EDMAB (2.5–100 μg/mL, R^2^ = 0.9999). Calibration curves were used to calculate the concentration of these species in the ethanol solutions; for example, the concentration was based on the chromatographic peak intensities corresponding to the retention times of MMeS (5.4 min), HEMA (9.3 min), MPS (10.2 min), EDMAB (29.0 min), and BisGMA (38.5 min).

### 4.7. Statistical Analysis

The results from the following experiments: water sorption, degree of conversion (FTIR), rate of polymerization, dynamic mechanical analysis (DMA), and accumulative concentration of leachates (HPLC) were analyzed using one-way analysis of variance (ANOVA) together with Tukey’s test at α = 0.05 (Microsoft Excel Microsoft 365, Microsoft Corporation. Redmond, WA USA). Statistical analyses were used to identify significant differences in means.

### 4.8. Adhesive/Dentin (a/d) Interface Preparation

Adhesive/dentin (a/d) interface specimens were prepared according to published protocols [53]. The extracted, de-identified human molars were stored at 4 °C in phosphate-buffered saline (PBS) containing 20 ppm sodium azide. These teeth, extracted for treatment purposes and collected from area oral surgeons, were anonymized, and the University of Kansas Institutional Review Board (IRB) approved their use in this research as exempt from IRB review.

To prepare the samples, the occlusal one-third of each molar’s crown was sectioned perpendicular to the tooth’s long axis using a water-cooled diamond disc mounted on a Isomet 1000 Precision Saw. The exposed dentin surfaces were etched for 90 s with 35% phosphoric acid, rinsed with Milli-Q water, and lightly air-dried to remove the excess superficial water. This etching protocol was intentionally more aggressive than typical clinical protocols to induce pronounced dentin demineralization.

The etched dentin surfaces were randomly assigned to be treated with either SC4 or SC5 adhesive formulation. SC4-treated samples received an additional NE1 treatment, whereas SC5-treated samples were treated with SC3. Polymerization was performed using a handheld Spectrum 800 unit with an irradiance of 550 mW/cm^2^ for 120 s, followed by dark curing in PBS at room temperature for a minimum of 48 h. The polymerized dentin surfaces were subsequently sectioned both perpendicular and parallel to the adhesive interface.

The a/d interface specimens were then characterized using micro-Raman spectroscopy to investigate the distribution of the adhesive, collagen (demineralized dentin), and mineral phases. A LabRAM ARAMIS Raman spectrometer (Horiba Jobin Yvon, Piscataway, NJ, USA) was used with a HeNe laser (λ = 633 nm, 17 mW laser power) and a 60× water-immersion objective lens (Olympus, Center Valley, PA, USA). The instrument settings included a 150 µm entrance slit, 200 µm confocal hole, 600 g/mm grating, and a spectral acquisition time of 10 s, with four acquisitions per cycle. The samples were mounted on a computer-controlled x-y positioning stage, and the Raman spectra were acquired over a range of 300–1800 cm^−1^. Two-dimensional micro-Raman mapping and imaging were used to determine the spatial relationships between functional groups. This approach followed the process described in our recent publication [41]. The samples were mapped by scanning in both the x- and y-directions. The scan was conducted over a grid with a distance of 1 micrometer between points on the x-axis and 2.5 micrometers between points on the y-axis. Ten lines were scanned vertically, consisting of 30 points horizontally, covering the designated area on the sample. This arrangement provided a detailed mapping of the surface of the sample, allowing for precise analysis of the scanned region. The acquired spectra were submitted for data processing using the LabSPEC 6 software (HORIBA Jobin Yvon, Piscataway, NJ, USA).

Data analysis incorporated several multivariate chemometric methods available in LabSpec 6′s Multivariate analysis (MVA) module (Horiba Jobin Yvon, Piscataway, NJ, USA). Among the available methods, classical least-squares (CLS), multivariate curve resolution (MCR), and divisive clustering analysis (DCA) were employed in this study.

Classical least-squares (CLS) fitting was employed to quantify the concentrations of known components within the spectra. This method utilizes reference spectra to model the acquired data, allowing the precise determination of component concentrations. By applying CLS, the spatial distribution and relative abundance of adhesive components in dentin can be effectively mapped. Such maps were prepared for both sample groups by employing the dentin and resin reference spectra. Multivariate curve resolution (MCR) was used to decompose the spectral data, enabling a detailed understanding of the distribution of individual components within the Raman maps. MCR allows for the resolution of pure component spectra from mixed data, facilitating the identification and quantification of the chemical species present in the samples. The acquired spectra were classified and grouped using three component divisive clustering analysis (DCA). An iterative process is used to divide the spectra into a specified number of groups such that the spectra belonging to the same group are similar to each other. The results of this iterative process were the class membership for each spectrum. The average spectrum of each class was displayed using statistical analysis. Using DCA, the average spectra were calculated for each class and these results were used to determine the component distribution in the Raman images.

## 5. Conclusions

Our results demonstrated that the chain length between the silane and methacrylate groups affected the thermal stability, hydrolytic resistance, and mechanical strength of the formulations with 30 wt% BisGMA. At the 30 wt% BisGMA-crosslinker concentration, the short-chain monomer formulations provided greater thermal stability and mechanical strength. With the exception of the HEMA leachate, the short-chain monomer formulations provided greater hydrolytic resistance. At 55 wt% BisGMA, the effect of chain length on mechanical strength was particularly pronounced at elevated temperatures under wet conditions, with the short-chain monomer showing threefold greater storage modulus values. In contrast, the chain length between the silane and methacrylate groups had no effect on the thermal stability or hydrolytic resistance of the formulations with 55 wt% BisGMA crosslinker.

The chain length between the silane and methacrylate groups led to pronounced differences in crosslink density and heterogeneity. The calculated crosslink densities of the short-chain monomer formulations with 30 wt% BisGMA were higher than those of the longer-chain monomers at the same crosslinker concentration. In addition, the calculated crosslink densities of the short-chain monomer formulations with 30 wt% BisGMA were higher than those with 55 wt% BisGMA. The short-chain monomer formulations with 30 wt% BisGMA showed significantly greater heterogeneity than the longer-chain monomer formulations at the same crosslinker concentration.

While the results are promising, further investigation is required to refine and optimize adhesive formulations. For example, the impact of phase separation on the a/d interfacial integrity requires rigorous analysis under conditions relevant to the mouth. Similarly, additional investigations are required to unravel the complex relationships between heterogeneity and mechanical behavior under dynamic loading.

## Figures and Tables

**Table 1:** Values of degree of conversion (DC%), maximum polymerization rate (R_p_^max^/[M]), and water sorption (W_sp_%).

Formulation	DC%	R_p_^max^ (1/[M])	W_sp_%
SC1	65.4 ± 0.7	12.5 ± 1.6	6.37 ± 0.5
SC2	63.9 ± 0.2	9.6 ± 2.3	6.76 ± 0.1
SC3	64.5 ± 0.2	11.7 ± 1.3	6.90 ± 0.1
SC4	79.7 ± 0.2	2.1 ± 0.2	14.23 ± 0.1
SC5	75.6 ± 0.2	2.8 ± 0.1	14.38 ± 0.5
NE1 [32]	65.7 ± 0.8	11.1 ± 2.8	7.28 ± 0.5

**Table 2:** Storage modulus, rubbery modulus, and glass transition temperature of vacuum-dried samples at various temperatures.

Formulation	Storage Modulus (GPa)	Rubbery Modulus (GPa)	T_g_ (°C)
SC1	3.96 ± 0.28 (37 °C)	0.20 ± 0.02	144.18 ± 5.9
	3.00 ± 0.20 (70 °C)		
SC2	3.97 ± 0.10 (37 °C)	0.08 ± 0.00	114.13 ± 2.5
	2.09 ± 0.16 (70 °C)		
SC3	4.24 ± 0.35 (37 °C)	0.27 ± 0.06	135.23 ± 13.6
	3.15 ± 0.28 (70 °C)		
SC4	4.21 ± 0.24 (37 °C)	0.10 ± 0.01	159.58 ± 0.8
	3.47 ± 0.19 (70 °C)		
SC5	4.40 ± 0.18 (37 °C)	0.36 ± 0.02	170.31 ± 0.9
	3.62 ± 0.15 (70 °C)		
NE1 [32]	3.85 ± 0.20 (37 °C)	0.20 ± 0.02	159.80 ± 4.6
	3.05 ± 0.08 (70 °C)		

**Table 3:** Storage modulus values under submerged conditions at various temperatures.

Formulation	Storage Modulus (GPa) at 37 °C	Storage Modulus (GPa) at 70 °C
SC1	2.14 ± 0.20	0.69 ± 0.12
SC2	1.95 ± 0.28	0.46 ± 0.16
SC3	2.34 ± 0.12	1.02 ± 0.13
SC4	1.14 ± 0.08	0.32 ± 0.03
SC5	1.49 ± 0.05	0.57 ± 0.02
NE1 [32]	1.74 ± 0.10	0.36 ± 0.03

**Table 4:** Values of the calculated crosslink density (ζ) and full width at half maximum (FWHM).

Formulation	Crosslink Density (ζ)	FWHM
SC1	2.32 ± 0.2 × 10^−6^ Pa^−1^ K	86.72 ± 5.2
SC2	5.61 ± 0.3 × 10^−6^ Pa^−1^ K	64.51 ± 2.8
SC3	1.69 ± 0.3 × 10^−6^ Pa^−1^ K	96.23 ± 5.8
SC4	1.97 ± 0.1 × 10^−6^ Pa^−1^ K	48.62 ± 1.1
SC5	0.52 ± 0.0 × 10^−6^ Pa^−1^ K	66.06 ± 2.1
NE1 [32]	2.20 ± 0.0 × 10^−6^ Pa^−1^ K	64.46 ± 6.0

**Table 5:** HEMA Leachates for SC3, SC4 and SC5 in prewash water.

Formulation	HEMA Leachate in Prewash (μg/mL)	HEMA Leachate in Prewash (wt%)
SC3	20.66 ± 1.74	0.30 ± 0.02
SC4	40.62 ± 0.02	0.31 ± 0.00
SC5	27.16 ± 0.84	0.20 ± 0.01

**Table 6:** Average cumulative leachate concentrations of components from SC3, SC4, and SC5 on day 13 compared with NE1.

Formulation	HEMA (μg/mL)	EDMAB (μg/mL)	BisGMA (μg/mL)
SC3	43.87 ± 4.0	Not detected	6.00 ± 0.9
SC4	40.57 ± 1.5	31.86 ± 1.6	5.01 ± 0.6
SC5	56.49 ± 2.2	22.22 ± 1.0	1.44 ± 0.2
NE1 [32]	50.42 ± 3.4	17.28 ± 1.1	17.56 ± 1.3

**Table 7:** Average cumulative leachate weight percentages of components from SC3, SC4, and SC5 on day 13 compared with NE1.

Formulation	HEMA (wt%)	EDMAB (wt%)	BisGMA (wt%)
SC3	0.63 ± 0.1	Not detected	0.04 ± 0.0
SC4	0.31 ± 0.0	25.49 ± 1.3	0.07 ± 0.0
SC5	0.43 ± 0.0	17.78 ± 0.8	0.02 ± 0.0
NE1 [32]	0.72 ± 0.1	13.82 ± 0.9	0.13 ± 0.0

**Table 8:** Chemical composition of the formulations.

Ratio%	SC1	SC2	SC3	SC4	SC5	NE1 [32]
HEMA	28	28	28	53	53	28
BisGMA	55	55	55	30	30	55
MPS	5	10	0	15	0	15
MMeS	10	5	15	0	15	0
3-PI ^1^	2	2	2	2	2	2
Total	100	100	100	100	100	100

^1^ Photoinitiators-CQ-EDMAD-DPIHP:0.5-0.5-1.

## Data Availability

All data cited in this study are publicly available.
